# Extremely Widespread Parthenogenesis and a Trade-Off Between Alternative Forms of Reproduction in Mayflies (Ephemeroptera)

**DOI:** 10.1093/jhered/esaa027

**Published:** 2020-09-12

**Authors:** Maud Liegeois, Michel Sartori, Tanja Schwander

**Affiliations:** 1 Department of Ecology and Evolution, University of Lausanne, Lausanne, Switzerland; 2 Cantonal Museum of Zoology, Palais de Rumine, Place de la Riponne, Lausanne, Switzerland

**Keywords:** mayflies, parthenogenesis, natural populations, evolution of sex

## Abstract

Studying alternative forms of reproduction in natural populations is of fundamental importance for understanding the costs and benefits of sex. Mayflies are one of the few animal groups where sexual reproduction co-occurs with different types of parthenogenesis, providing ideal conditions for identifying benefits of sex in natural populations. Here, we establish a catalog of all known mayfly species capable of reproducing by parthenogenesis, as well as species unable to do so. Overall, 1.8% of the described species reproduce parthenogenetically, which is an order of magnitude higher than reported in other animal groups. This frequency even reaches 47.8% if estimates are based on the number of studied rather than described mayfly species, as reproductive modes have thus far been studied in only 17 out of 42 families. We find that sex is a more successful strategy than parthenogenesis (associated with a higher hatching success of eggs), with a trade-off between the hatching success of parthenogenetic and sexual eggs. This means that improving the capacity for parthenogenesis may come at a cost for sexual reproduction. Such a trade-off can help explain why facultative parthenogenesis is extremely rare among animals despite its potential to combine the benefits of sexual and parthenogenetic reproduction. We argue that parthenogenesis is frequently selected in mayflies in spite of this probable trade-off because their typically low dispersal ability and short and fragile adult life may frequently generate situations of mate limitation in females. Mayflies are currently clearly underappreciated for understanding the benefits of sex under natural conditions.

The evolution and maintenance of sexual reproduction has been one of the major questions in evolutionary ecology for the last decades (e.g., Agrawal 2006; Otto 2009; Jalvingh et al. 2016). Sex is associated with profound costs (reviewed in Lehtonen et al. 2012), yet it is the most widespread reproductive mode among animals. Female-producing parthenogenesis (thelytoky) would largely avoid the costs associated with sex, yet only a minority of animals are known to reproduce parthenogenetically. The proportion of parthenogenetic species remains however unknown as there are only 2 quantitative estimates (based on species lists) of the frequency of parthenogenesis (i.e., in vertebrates: White 1973; Vrijenhoek 1998; and in haplodiploids: van der Kooi et al. 2017). This is unfortunate as such species lists are invaluable for addressing when and how parthenogenetic reproduction is favored over sex in natural populations (e.g., Ross et al. 2013; van der Kooi et al. 2017) and thus for helping to solve the paradox of sex. In this review, we provide such a quantitative estimate by summarizing the current knowledge on sexual and parthenogenetic reproduction in Ephemeroptera (mayflies), as a first step towards developing this group for the study of benefits of sex in natural populations.

Mayflies constitute one of the most basal (early-diverging) lineages of insects (Edmunds and McCafferty 1988), their origin dating back to ~300 Mya (Brittain and Sartori 2009). Widespread around the world with 3,666 described species (42 families, 472 genera; adapted from Sartori and Brittain 2015; MS, unpublished), they are mostly studied for fly fishing purposes (Knopp and Cormier 1997), and for their potential as bioindicators of water quality (Bauernfeind and Moog 2000). Mayflies do not feed as adults, relying solely on the energy reserves accumulated during their aquatic larval stages. Adult life span is extremely short, lasting from few hours to some days, depending on the species. Because of their typically low dispersal ability and their short and fragile adult life, mayflies have restricted opportunities for reproduction, which we argue is one of the factors that may have selected for the evolution of parthenogenesis in this group. Parthenogenesis in mayflies can be largely accidental (i.e., tychoparthenogenesis), facultative or “obligate” (Box 1). Furthermore, a single species can feature mixed reproduction (some females reproduce sexually, others parthenogenetially), either sympatrically or in allopatry (i.e., geographical parthenogenesis).

**Box 1. Three forms of female-producing (thelytokous) parthenogenesis in mayflies**

(A) Tychoparthenogenesis or “spontaneous parthenogenesis,” occurs in sexual species (typically less than 10% of unfertilized eggs develop through parthenogenesis). Given the low hatching success of unfertilized eggs, population sex ratios remain equal. (B) Facultative parthenogenesis, means that eggs may either be fertilized or develop parthenogenetically. The hatching success of unfertilized eggs, in this case, is intermediate (typically 10–75%), leading to female-biased population sex ratios. (C) Under “obligate” parthenogenesis, eggs always develop parthenogenetically and likely cannot be fertilized, with a hatching success typically higher than 75%. Only females are present in these populations. Note that an individual species c an feature multiple forms of parthenogenesis, in the same or different populations.

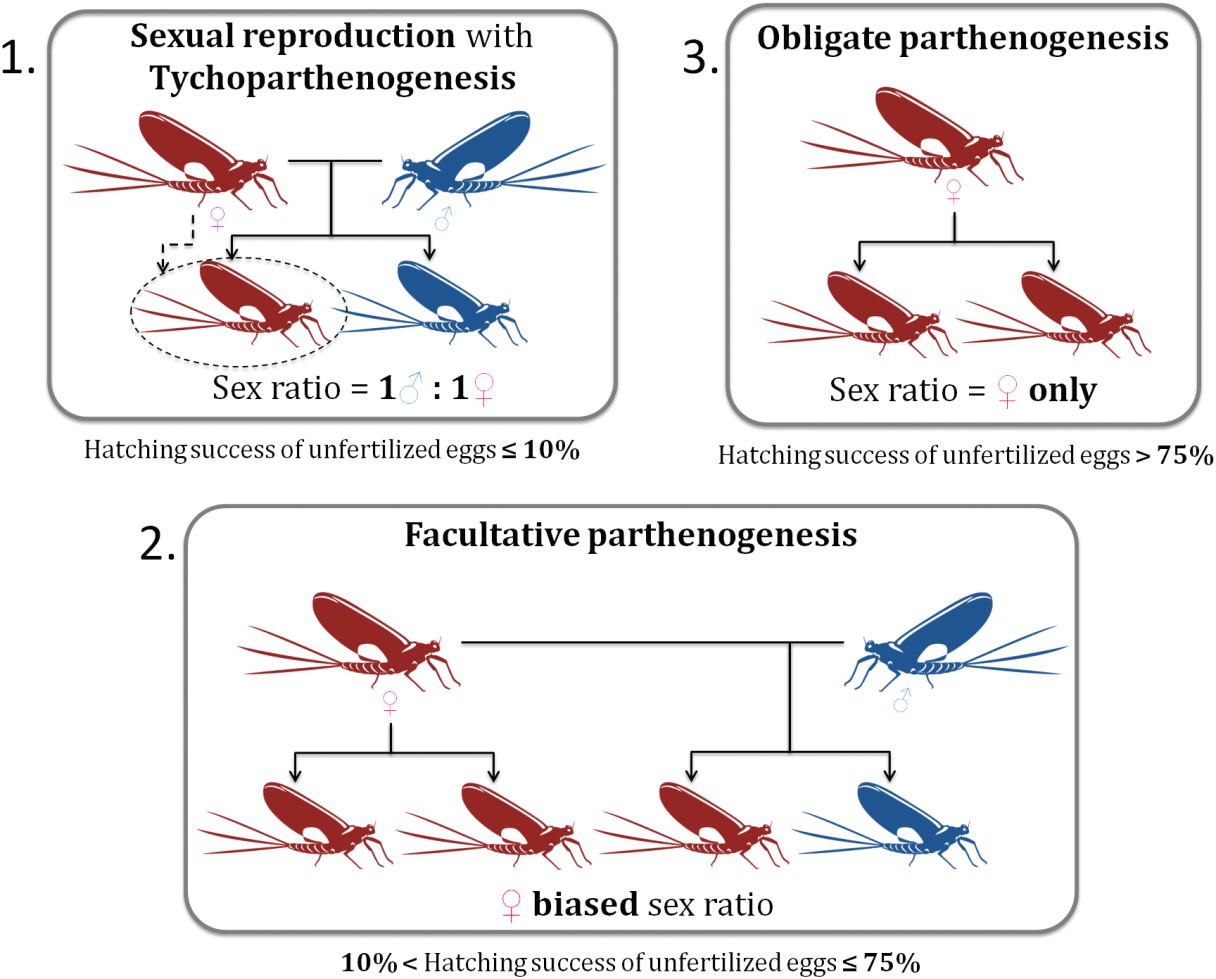

We conducted a detailed literature review to establish a catalog of all (to the best of our knowledge) mayfly species capable of reproducing parthenogenetically (Supplementary Appendix). We then used this catalog to study whether the frequency of parthenogenesis varies among mayfly clades, in order to evaluate how parthenogenesis evolves in this group, and whether it tends to be phylogenetically clustered. We conducted cross-species comparisons with respect to the cellular mechanisms of parthenogenesis and the hatching success of fertilized and unfertilized eggs, to test whether the abilities of reproducing parthenogenetically or sexually are negatively correlated. We also looked at how the capacity for parthenogenesis affects population sex ratios, as female-producing parthenogenesis should bias the population towards females (Box 1). Finally, we compared the geographical distribution of sexual and parthenogenetic populations to detect large- and small-scale patterns of geographical parthenogenesis.

## Material and Methods

### Data Collection

The species list was compiled by collecting information from a thorough search of the literature, using Google Scholar, and Web of Science (publications until August 2019), as well as Ephemeroptera Galactica (www.ephemeroptera-galactica.com) and Ephemeroptera of the world (www.insecta.bio.spbu.ru/z/Eph-spp/Contents.htm). Four previous reviews (Degrange 1960; Humpesch 1980; Sweeney and Vannote 1987; Funk et al. 2010) comprised 78 mayflies species studied for their reproductive mode. The Tree of Sex database (Ashman et al. 2014) comprised only 8 sexual and one parthenogenetic mayfly species and was therefore not useful for our survey. Our survey combined with personal communications and observations generated a list of 136 species, as described in our database (Table 1, Supplementary Appendix). When available for a given species, information on its geographical distribution, cytological mechanism of parthenogenesis, and sex determination was included in the database (Supplementary Appendix).

**Table 1.** Frequency of parthenogenesis and sex determination in Ephemeroptera taxa

				% of parthenogenesis			
Family	Parthenogenetic species	Sexual species	Total number of described species	Among described species	Among studied species	Sex determination (2n+♀:♂)	References
Ameletidae	4	1	60	6.7	80.0	16+XX:XY	Morgan 1911; Clemens 1922; Needham 1924; Traver 1932; Katayama 1939; Wolf 1946; Burks 1953; Sweeney and Vannote 1982, 1987; Jazdzewska and Wojcieszek 1997; Funk et al. 2006; Reding 2006
Baetidae	35	10	1076	3.3	77.8	8+XX:XY	Bengtsson 1913; Dodds 1923; McDunnough 1925, 1936; Ide 1930; Degrange 1954, 1955, 1956, 1960; Tjønneland 1960; Wolf 1960; Hirvenoja 1964; Uéno 1966; Bohle 1969; Gibbs 1977; Kiauta and Mol 1977; Bergman and Hilsenhoff 1978; McCafferty and Morihara 1979; Kluge 1980; Harper and Harper 1984; Sweeney and Vannote 1984, 1987; Sivaramakrishnan et al. 1991; Lowen and Flannagan 1992; Sweeney et al. 1993; Jackson and Sweeney 1995; Harker 1997; Salas and Dudgeon 1999; Gattolliat and Sartori 2000; Soldan and Putz 2000; Funk et al. 2006, 2008, 2010; Encalada and Peckarsky 2007; Giberson et al. 2007; Webb et al. 2012; Martynov 2013; ML, personal observation
Baetiscidae	-	1	12	0	0	NA	Pescador 1973; Pescador and Peters 1974
Behningiidae	-	1	7	0	0	NA	Peters and Peters 1977; Harvey et al. 1980; Sweeney and Vannote 1982
Caenidae	5	4	258	1.9	55.6	6+XX:XO	Degrange 1960; Froehlich 1969; Mol 1978; Gillies and Knowles 1990; Da-Silva 1993, 1998; Hofmann et al. 1999; Soldan and Putz 2000; Malzacher and Staniczek 2007; FFS, personal communication
Dipteromimidae	-	1	2	0	0	NA	Takenaka et al. 2019
Ephemerellidae	7	4	154	4.5	63.6	6, 14+XX:XY	Needham 1924; Degrange 1960; Kazlauskas 1963; Fiance 1978; Mol 1978; Harper and Harper 1982; Sweeney and Vannote 1987; Soldan and Putz 2000; Glazaczow 2001; Funk et al. 2008, 2010; Webb et al. 2012
Ephemeridae	1	10	85	1.2	9.1	10, 12+XX:XO	Neave 1932; Wolf 1946; Hunt 1951; Degrange 1960; Britt 1962; Thomforde and Fremling 1968; Friesen and Flannagan 1976; Mol 1978; Sweeney and Vannote 1987; Soldan and Putz 2000; Funk et al. 2010; Sekiné and Tojo 2010a
Heptageniidae	3	22	610	0.5	12	18+XX:XY	Degrange 1955, 1960; Huff and McCafferty 1974; McCafferty and Huff 1974; Mingo 1978; Mol 1978; Humpesch 1980; Salas and Dudgeon 1999; Soldan and Putz 2000; Ball 2001, 2002; Funk et al. 2010; ML, personal observation
Isonychiidae	-	1	35	0	0	NA	Sweeney and Vannote 1987
Leptophlebiidae	4	10	726	0.6	28.6	12–14+XX:XY	Degrange 1960; Clifford et al. 1979; Savage 1986; Sweeney and Vannote 1987; Jackson and Sweeney 1995; Salas and Dudgeon 1999; Soldan and Putz 2000; Funk et al. 2010
Oligoneuriidae	-	1	66	0	0	14+XX:XY	Degrange 1960; Soldan and Putz 2000
Palingeniidae	1	-	33	3	100	NA	Landolt et al. 1997
Polymitarcyidae	2	2	104	1.9	50	10, 14+XX:XO	Degrange 1960; Britt 1962; Tojo et al. 2006; Sekiné and Tojo 2010b, 2010a; Sekiné et al. 2013, 2015
Potamanthidae	-	1	25	0	0	6+XX:XO	Degrange 1960; Soldan and Putz 2000
Prosopistomatidae	1	1	29	3.4	50	NA	Degrange 1960; Campbell and Hubbard 1998
Siphlonuridae	2	1	48	4.2	66.7	16+XX:XY	Degrange 1954, 1955, 1960; Gibbs and Siebenmann 1996; Soldan and Putz 2000
Total	65	71^a^	3,666	1.8%	47.8%		

Only families with at least one species studied for its reproductive mode are shown (17 families out of 42 [40.5%] have been studied). The numbers of described species have been adapted from Sartori and Brittain (2015) and updated (MS, unpublished). NA: unknown. Note that all studied sexual and parthenogenetic species are diploid (2n).

^a^Among the 71 sexual species, 38 can reproduce by tychoparthenogenesis (53.5%).

### Classification of Parthenogenesis

To classify mayfly species by forms of parthenogenesis (Box 1), we focused on the hatching rate of unfertilized eggs. We defined 2 main categories according to this rate: “sexual species” (including species with tychoparthenogenesis), when less than 10% of unfertilized eggs hatch (typically 0–5.3% for population means), and “parthenogenetic species,” when hatching success of unfertilized eggs is higher than 10% (typically 18.5–97.3% for population means). Parthenogenetic species include all species with facultative parthenogenesis, “obligate” parthenogenesis, and mixed reproduction. Note that species with mixed reproduction have low population-average hatching success of unfertilized eggs when sexual and parthenogenetic females occur in sympatry (see Results). We also considered species as parthenogenetic if female-only populations were reported in the literature, even if these species were not directly tested for their parthenogenetic capacity (this was the case for 18 of the 136 species in our database).

Within parthenogenetic species, we further distinguished “obligate” from facultative parthenogens. Excluding rare events of sex in putatively obligate pathenogens is difficult (reviewed in Schurko et al. 2009). We used the term obligate parthenogens for species where no males are known, or where rare males (typically <0.1% of all individuals) are most likely vestiges of sexual reproduction (van der Kooi and Schwander 2014), indicating that parthenogenesis is the main form of reproduction. We also found very rare mentioning of deuterotoky (where both males and females are produced parthenogenetically). Specifically, the baetid species *Centroptilum luteolum*, *Acerpenna pygmaea*, *Acerpenna macdunnoughi*, and *Anafroptilum semirufum* were inferred to be deuterotokous in breeding studies (Degrange 1956; Funk et al. 2010) because parthenogenetic broods contained high frequencies (2–17%) of males. Occasional deuterotoky is also the most likely explanation for the occurrence of rare males in “obligately” parthenogenetic species as mentioned above. It is currently unclear how such males are produced (e.g., via environmental influences on sex determination or X-chromosome losses in species with XX/X0 sex determination). The few known deuterotokous species are counted as parthenogenetic species in our classification.

### Quality of the Data and Limitations of the Database

Our database includes the species for which information concerning their reproductive mode is available. Of course, research efforts are not homogeneously distributed among the different taxa or different geographic ranges, which constraints the conclusions one can draw from the available information. For example, there is a strong study bias for species in the Northern Hemisphere, which precludes broad geographic comparisons for the distribution of sexual and parthenogenetic species (see the section on geographical parthenogenesis).

There is also a study bias for certain taxa, with 70 species in our database belonging to only 2 families (Baetidae and Heptageniidae; Table 1). In the Baetidae family, 45 species have been studied for their reproductive mode, which represent one-third of the species in our database (Table 1, see Supplementary Appendix for details). However, this family is also the most species-rich one (with 1076 = 29.4% of the taxonomically described species; Table 1). The Heptageniidae family contributes 25 species to our database, which, again, largely reflects its taxonomic richness (610 = 16.6% of the taxonomically described species).

Nevertheless, in order to account for these taxonomic biases, we repeated our statistical analyses based on our database after excluding these 2 families (using solely the species of the 15 remaining families; Table 1). Excluding the 2 families from the analyses did not change any of our main conclusions (see below).

### Statistical Analyses

**Figure 1.**
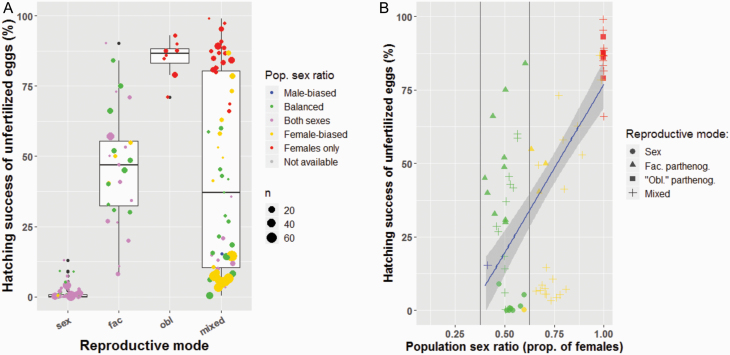
(**A**) Population-average hatching success of unfertilized eggs and reproductive modes. *x* axis: sex: sexual reproduction, fac: facultative parthenogenesis, obl: “obligate” parthenogenesis, mixed: mixed reproduction (in sympatry and/or in allopatry). Pop. sex ratio: population sex ratio. Both sexes (purple dots) means that males are present in these populations, but exact sex ratios were not recorded; *n*: number of females used for determining the egg-hatching success in a given population. In total, 186 populations from 108 different species are represented (with species from 17 families but mostly Baetidae (32.3%) and Heptageniidae (27.4%), see also Supplementary Figures 1 and 2). (**B**) Population-level correlation between unfertilized egg-hatching success and sex ratio (GLMM, *r* = 0.72, *P*-value < 0.001). Sex: sexual reproduction, Fac. parthenog.: facultative parthenogenesis; “Obl.” parthenog.: “obligate” parthenogenesis, Mixed: mixed reproduction (in sympatry and/or in allopatry). Data are available for 84 populations from 31 species (with species from 11 families but mostly Baetidae (23.8%) and Heptageniidae (20.2%), see also Supplementary Figures 3 and 4).

We first verified that our classification into sexual (with or without tychoparthenogenesis) and parthenogenetic species (facultative, mixed, and obligate) was biologically meaningful, by comparing the distribution of hatching successes of unfertilized eggs for different groups (Figure 1A). We further tested whether species with high egg-hatching successes were often characterized by female-biased population sex ratios, by using a quasibinomial Generalized Linear Mixed Model (GLMM) with the R v.3.3.3 (R Development Core Team 2017) “MASS” (Venables and Ripley 2002) and “car” (Fox and Weisberg 2011) packages. To account for population means based on low numbers of individuals, we weighted our analyses with the number of females tested for each population.

We compared the prevalence of parthenogenetic species among mayfly families, using the most recent phylogeny available (Ogden et al. 2019). Lack of phylogenetic information at lower taxonomic levels precluded further analyses. We thus tested whether variations in the frequency of parthenogenesis among families were explained by their phylogenetic relatedness by using binomial Generalized Linear Models (GLMs) and Tukey tests with the “multcomp” (Hothorn et al. 2008) package. In addition, we tested whether the frequency of parthenogenesis in mayflies varies among the 6 broad geographical regions: Nearctic, Palearctic, Neotropical, Afrotropical, Oriental, and Australasian (see map in Table 2). Finally, we tested for potential trade-offs between parthenogenetic and sexual reproduction by studying hatching rate of fertilized and unfertilized eggs at the population level of a given species (Figure 5).

**Table 2.**
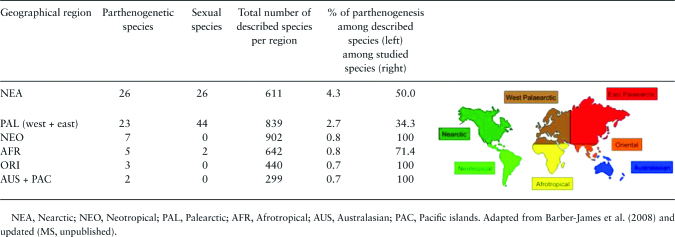
World distribution of parthenogenesis in Ephemeroptera

NEA, Nearctic; NEO, Neotropical; PAL, Palearctic; AFR, Afrotropical; AUS, Australasian; PAC, Pacific islands. Adapted from Barber-James et al. (2008) and updated (MS, unpublished).

A key aim of our study was to estimate the frequency of parthenogenesis among mayfly species. Estimating the frequency of parthenogenetic species in a taxon is difficult because the reproductive mode for the vast majority of species has not been studied. The 2 available previous estimates of the frequency of parthenogenesis among animals (in vertebrates: White 1973; Vrijenhoek 1998; and haplodiploid insects: van der Kooi et al. 2017) assumed that all described species without evidence for parthenogenesis were sexual. However, this assumption severely underestimates the frequency of parthenogenesis. To account for this underestimation, we generated 2 frequency estimates, one using the total number of taxonomically described mayfly species, and one using only species where the reproductive mode was studied.

## Results

### Unfertilized Egg-Hatching Successes and Population Sex Ratios

Analyzing the information we collected in our database revealed that the parthenogenetic capacity of females varied widely between and within populations (see Supplementary Appendix for details). Nevertheless, our classification into sexual (with or without tychoparthenogenesis) and parthenogenetic species (facultative or obligate) is biologically meaningful, given the largely non-overlapping values for population-average hatching success of unfertilized eggs (Figure 1). Note that some species with mixed reproduction show a low population-average hatching success of unfertilized eggs when sexual and parthenogenetic females occur in sympatry (e.g., average of 5.7% for one population of *Stenonema femoratum*, with egg-hatching successes varying among females from 0 to 77.9%). In order to determine whether a higher capacity for parthenogenesis translates into female-biased population sex ratios, we used species where both sex ratios and unfertilized egg-hatching successes were studied in the same populations. In these species, the parthenogenetic capacity and population sex ratios were significantly positively correlated (Figure 1B, *GLMM, r* = 0.72, *P*-value < 0.001). The parthenogenetic capacity of females in strongly biased populations (>60% of females) was always very high (median: 83.4%, range: 40.4–97.3%), except for the species with mixed reproduction in sympatry as mentioned above (median: 7.8%, range: 3.3–15.5%). Conversely, unbiased population sex ratios were not indicative of species with obligate sexual reproduction, as they frequently comprised females with a high parthenogenetic capacity (Figure 1).

### Frequency of Parthenogenesis Among Mayflies

**Figure 2.**
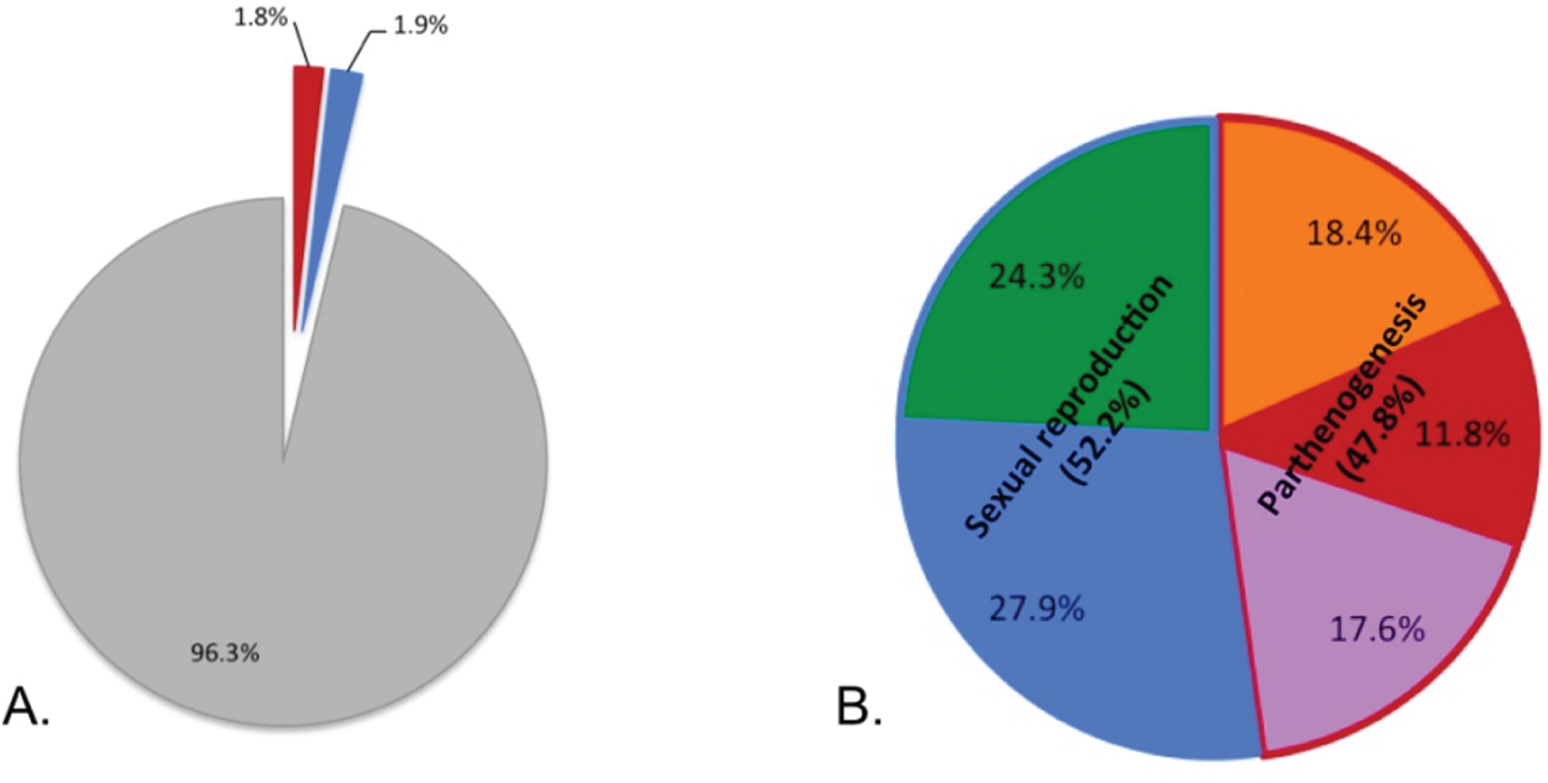
Frequency of parthenogenesis among described mayfly species (**A**) and among species studied for their reproductive mode (**B**). Less than 4% of the mayfly species (from 17 out of 42 families) have been studied for their reproductive mode. Parthenogenesis (warm colors): facultative (orange), “obligate” (red) and mixed reproduction (purple). Sexual reproduction (cold colors): “obligate” sexual reproduction (green) and sexual reproduction with tychoparthenogenesis (blue). At least 47.8% of the 136 studied species are able to reproduce parthenogenetically.

**Figure 3.**
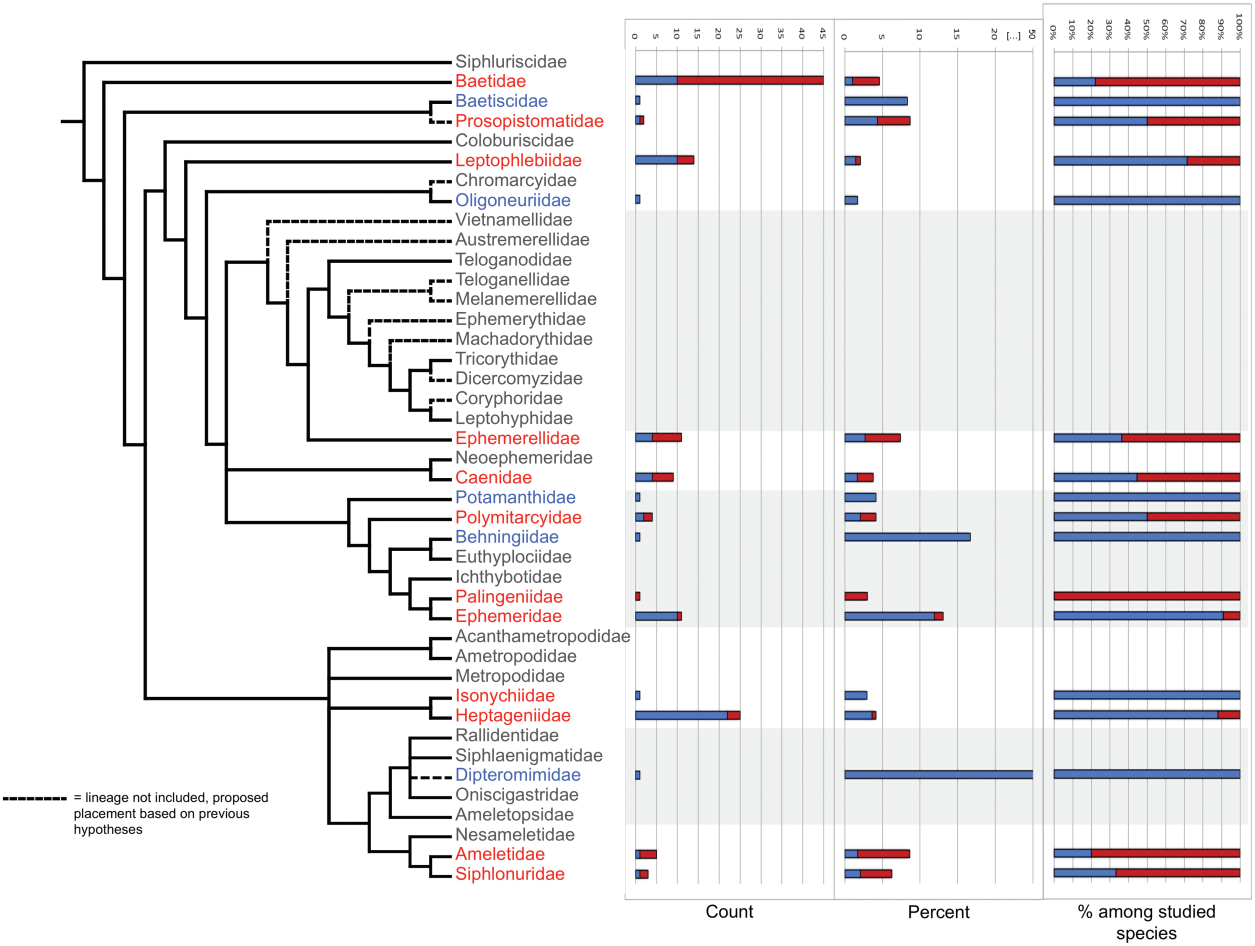
Phylogenetic distribution of parthenogenesis among mayfly families. red color: Parthenogenetic species (facultative, obligate, and mixed), blue color: Sexual species (with or without tychoparthenogenesis), gray color: Families without information regarding species’ reproduction (*n* = 25). The families Heptageniidae, Leptophlebiidae, and Ephemeridae show a low propensity for parthenogenesis, whereas the families Baetidae, Ameletidae, and Ephemerellidae show a high propensity for parthenogenesis (see main text for details). Count: number of mayfly species studied for their reproductive mode so far; Percent: percentage among the taxonomically described mayfly species in a given family; % among studied species: percentage among the mayfly species studied for their reproductive mode in a given family. Phylogeny adapted from Ogden et al. (2009, 2019). Note that we consider sexual reproduction (with tychoparthenogenesis) to be the ancestral state in mayflies.

Parthenogenesis occurs in all well-studied mayfly families (Table 1, Figure 3). We were able to classify the reproductive mode of 136 species from 17 families (Table 1, see Supplementary Appendix for details). Seventy-one of these species are sexual (from 16 families), and 38 of these are able to perform tychoparthenogenesis, while 65 species are parthenogenetic (from 11 families). Assuming the 3,666 described mayfly species without information concerning their reproductive mode are sexual, 1.8% of all mayfly species are able to reproduce parthenogenetically, which is at least an order of magnitude higher than the available estimate for vertebrates (0.1%, White 1973; Vrijenhoek 1998), and comparable to the frequencies in other arthropod orders. For example, the frequency of parthenogenesis in orders with haplodiploid sex determination varies from 0 to 1.5% (van der Kooi et al. 2017). However, if one uses the frequency estimates based on the number of mayfly species studied for their reproductive mode (*n* = 136), the estimated frequency of parthenogenesis reaches 47.8% (Figure 2), being about 25 times higher. These findings suggest that half of the mayfly species might be able to reproduce parthenogenetically, or even, that most mayflies are able to reproduce at least by tychoparthenogenesis (75.7% of the studied species).

Parthenogenesis occurs in an array of families and genera without any evidence for phylogenetic clustering (Figure 3). A similar pattern is observed in haplodiploid taxa (van der Kooi et al. 2017). These findings suggest that putative predispositions for the evolution of parthenogenesis do not have a strong phylogenetic inertia within the studied groups. However, clustering occurs at lower taxonomic levels as the proportion of parthenogenetic species varies significantly among mayfly families (G-tests of independence; studied species, *P* < 0.001; described species, *P* < 0.001). Indeed, parthenogenetic mayfly species are rarer in the families Heptageniidae, Leptophlebiidae and Ephemeridae (<1.5% among the described mayfly species, or <30% among the one studied for their reproductive mode), than in the families Baetidae, Ameletidae, and Ephemerellidae (>3.0% or >60%). In the remaining families, the data are too scarce to detect potential differences.

### Geographical Parthenogenesis

The term geographical parthenogenesis is used when parthenogenetic populations are found at higher altitudes or latitudes than their sexual counterparts, in harsher environmental conditions or have wider distributions and/or ecological niches (Vandel 1928). Such different distributions of sexual and asexual species could provide insights into ecological conditions that favor sex or parthenogenesis in natural populations, but quantitative comparisons are scarce (reviewed in Tilquin and Kokko 2016).

Considering very broad geographical scales and among-species comparisons, we found no evidence for the geographic clustering of parthenogenetic species. The 6 regions compared comprised approximately equal proportions of studied parthenogenetic species (Table 2, see Supplementary Appendix for details). However, even if such differences existed, we would not be able to uncover them with the currently available data. Indeed, among the 136 studied species, 117 come from Nearctic and Palearctic regions (86%), with very little data available for the remaining regions of the world.

Considering within-species comparisons at smaller geographical scales, there are at least 18 mayfly species (27.7% of the parthenogenetic species) with both sexual and parthenogenetic populations (see Supplementary Appendix for details). Two of them feature geographical parthenogenesis, but with distinct distribution differences between parthenogens and sexuals. Parthenogenetic populations of *Eurylophella funeralis* (Ephemerellidae) mostly occur at the periphery of the species ranges in North America (Sweeney and Vannote 1987), while parthenogenetic populations of *Ephemerella notata* (Ephemerellidae) occur at lower latitudes than sexual ones in Poland (Glazaczow 2001). One of the 18 species does not feature geographical parthenogenesis. Indeed, no geographical pattern is observed for *Ephoron shigae* (Polymitarcyidae), a species where sexual and parthenogenetic populations broadly overlap in Japan (Watanabe and Ishiwata 1997). Finally, for 15 of the 18 species where sexual and parthenogenetic populations are known to occur in separate geographical areas, the number of described populations is too small to identify a systematic distribution difference between sexual and parthenogenetic populations (Supplementary Appendix). Thus, the lack of data for these species does not allow us to conclude anything regarding potential geographical patterns.

More than the 2 discussed cases of geographical parthenogenesis likely exist in mayflies but are not detected because of a lack of studies, especially in the southern hemisphere. However, it seems that geographical parthenogenesis is not necessarily associated with particular ecological factors, as is the case in most other taxonomic groups studied thus far (Tilquin and Kokko 2016).

### Cytological Mechanisms of Parthenogenesis in Mayflies

**Figure 4.**
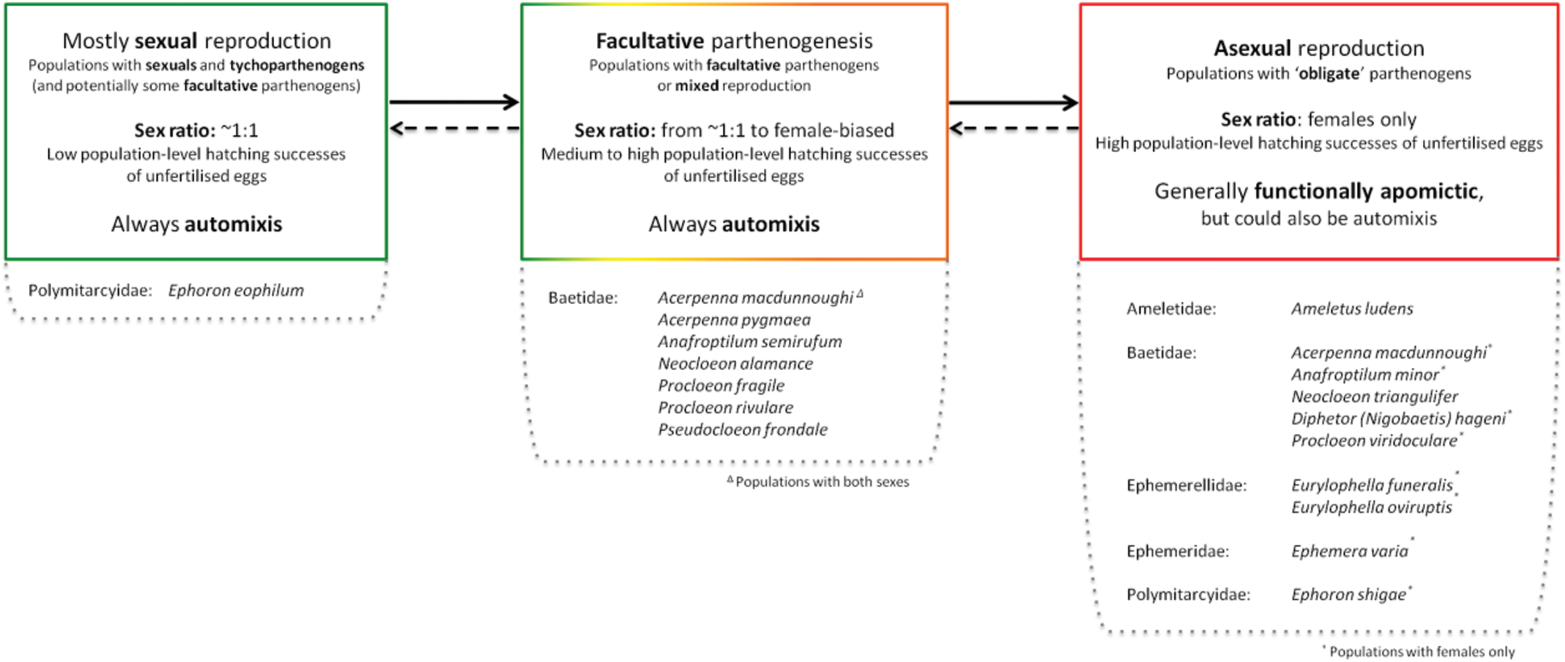
Cytological mechanisms identified to date in mayflies and possible transitions between parthenogenesis forms.

In animals, different cytological mechanisms can underlie thelytokous parthenogenesis, which vary with respect to their consequences on heterozygosity in offspring (reviewed in Suomalainen et al. 1987). In mayflies, some of these mechanisms have been identified or suggested, but studies remain scarce. Nevertheless, the available information suggests that obligate parthenogens use cytological mechanisms that potentially allow for maintenance of heterozygosity across generations (but see Jaron et al. 2018), while facultative parthenogens are invariably automictic and produce parthenogenetic offspring that are highly homozygous relative to sexual offspring. Specifically, 9 out of the 10 studied “obligately” parthenogenetic mayflies are functionally clonal without a detected loss of heterozygosity between generations (Sweeney and Vannote 1987; Sweeney et al. 1993; Funk et al. 2006, 2008, 2010). Three mechanisms can be responsible for the complete maintenance of heterozygosity between generations: apomixis (parthenogenesis is functionally mitotic), endoduplication, or automixis with central fusion (without recombination). Which one(s) of these mechanisms occur in mayflies is currently unknown. The cytological mechanism of the remaining species, *E. shigae* in Japan, was suggested to be automixis with terminal fusion (Sekiné and Tojo 2010b), indicating that some “obligate” parthenogens might not be clonal. All 7 studied facultatively parthenogenetic mayflies are automictic (Supplementary Appendix). Indeed, for the populations with both males and females of 7 Baetidae species (*Acerpenna macdunnoughi*, *A. pygmaea*, *Anafroptlilum semirufum*, *Labiobaetis frondalis*, *Neocloeon alamance*, *Procloeon fragile*, *P. rivulare*) parthenogenesis appears to be automictic with terminal or central fusion (with recombination), given the partial loss of heterozygosity between generations, but further details are not known (Funk et al. 2010). Finally, the cytological mechanism in *Ephoron eophilum* (Polymitarcyidae), a mostly sexual species with some facultatively parthenogenetic females (i.e., mixed reproduction in sympatry) is either automixis with terminal fusion (without recombination) or gamete duplication, where complete homozygosity is achieved in one generation (Sekiné et al. 2015). Cytological mechanisms of parthenogenesis in mayflies clearly require additional studies, but the major mechanisms identified to date are summarized in Figure 4.

### Trade-Off Between Reproductive Modes

**Figure 5.**
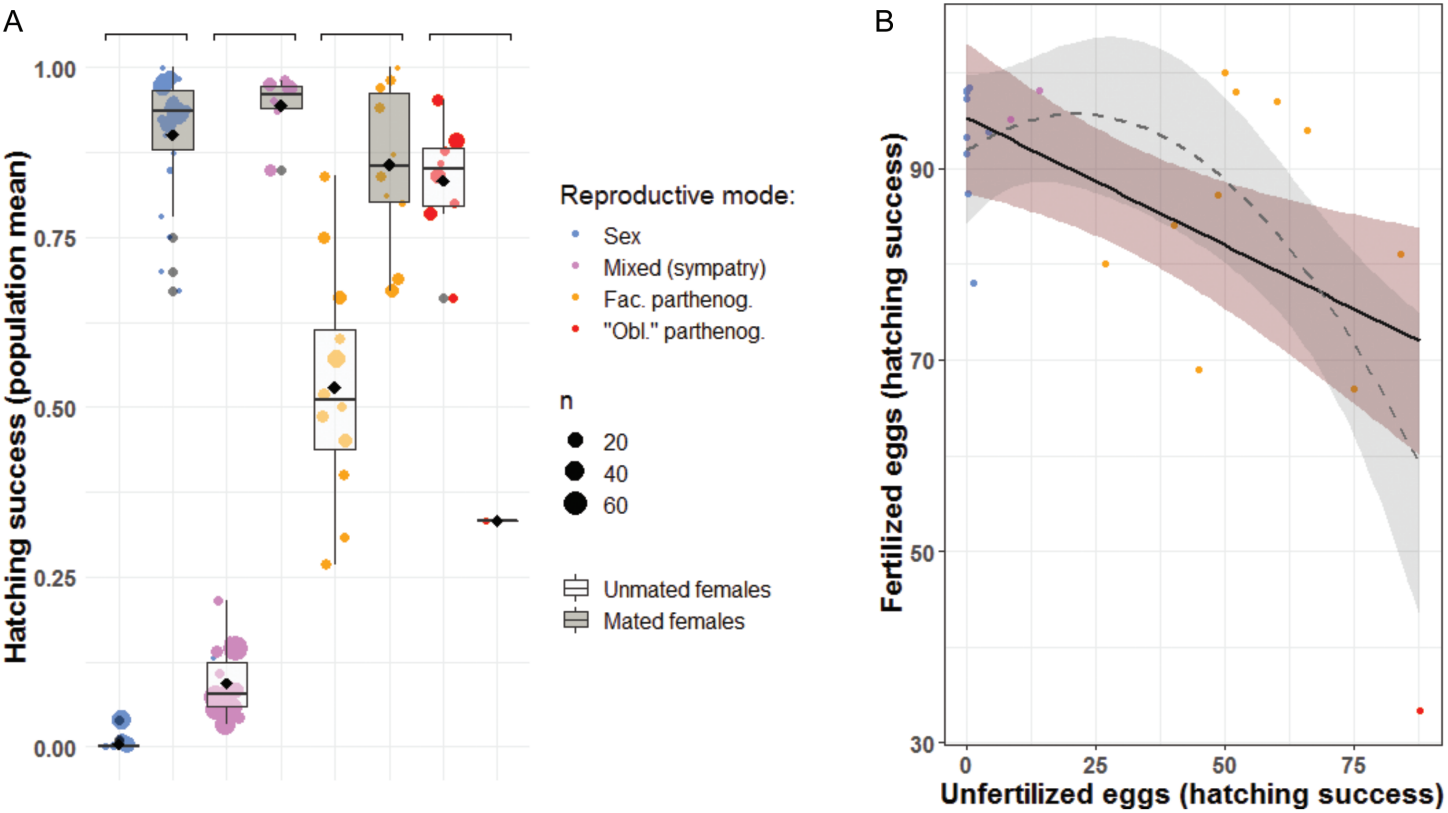
(**A**) Hatching success of fertilized and unfertilized eggs for species with different reproductive modes and mating status. Data are available for 75 populations from 32 different species, among 8 families (but mostly Baetidae (29.3%) and Heptageniidae (28.0%), see also Supplementary Figures 5 and 6). *n*: number of females tested for a given population. Sex: sexual reproduction (*P* < 0.001), Mixed: mixed reproduction in sympatry (*P* < 0.001), Fac. parthenog.: facultative parthenogenesis (*P* < 0.001), “Obl.” parthenog.: “obligate” parthenogenesis (*P* < 0.1); (**B**) Trade-off between parthenogenesis and sexual reproduction of a given population (GLMM, *r = −*0.50, *P*-value = 0.013). Each point represents one population for which hatching success of fertilized and unfertilized eggs were measured. Data are available for 22 populations from 19 species, among 7 families (but mostly Baetidae [45.4%] and Heptageniidae [9.1%]).

Overall, mayfly species are better at reproducing sexually than asexually (measured as egg-hatching success, Figure 5A, *P*-value < 0.001). Only in obligate parthenogens is egg-hatching success decreased upon mating, presumably because (even partial) fertilization interferes with the normal development of asexual eggs. Furthermore, there is a significant negative correlation between hatching rate of fertilized and unfertilized eggs at the population level of a given species (Figure 5B, GLMM, *r* = −0.50, *P*-value = 0.013). This negative correlation suggests that there are trade-offs between parthenogenetic and sexual reproduction, meaning that improving the capacity for parthenogenesis may come at a cost for sexual reproduction, even in facultative parthenogens. If such a trade-off indeed exists, it could help explain why facultative parthenogenesis is extremely rare among animals in spite of its potential to combine the benefits of sexual and parthenogetetic reproduction.

### Origins of “Obligate” Parthenogenesis in Mayflies

There are at least 4 ways in which parthenogenetic lineages could arise from sexual species in animals (Simon et al. 2003): 1) Hybridization between 2 sexual species, which is the major route to parthenogenesis in vertebrates (Avise et al. 1992); 2) Contagious origin from preexisting parthenogenetic lineages, where males produced by parthenogenetic species generate new lineages by mating with sexual females (e.g., in aphids Jaquiéry et al. 2014; and water fleas Xu et al. 2015); 3) Infection by microorganisms (e.g., *Wolbachia*, *Cardinium*, *Rickettsia*), mostly in species with haplodiploid sex determination (reviewed in Ma and Schwander 2017) and 4) Spontaneous transitions through mutations, for example, with tychoparthenogenesis as a first step (Carson et al. 1957; Kramer and Templeton 2001; Schwander and Crespi 2009; Schwander et al. 2010).

In mayflies, there is no evidence of parthenogenesis induced by hybridization or endosymbiont infection, but there is very little data informing on the origins of parthenogenesis. A hybrid origin seems unlikely because it usually results in high levels of heterozygosity (recently reviewed in Jaron et al. 2018), which is not the case for unisexual populations of the mayfly species studied so far (Sweeney and Vannote 1987; Funk et al. 2006; Sekiné and Tojo 2010b). Endosymbiont-induced parthenogenesis is also unlikely in mayflies because parthenogenesis in this group is often facultative, while endosymbiont infection normally causes obligate parthenogenesis (reviewed in Ma and Schwander 2017). In addition, sex determination is male heterogamety (not haplodiploidy, Table 1, see Supplementary Appendix for details), further reducing the probability for endosymbiont-induced parthenogenesis.

In mayflies, it has been speculated that facultative and obligate parthenogenesis originates from tychoparthenogenesis (Sweeney and Vannote 1987; Tojo et al. 2006). Although this is a plausible hypothesis given how widespread tychoparthenogenesis is among mayflies (27.9% of the studied species, Figure 2, see Supplementary Appendix for details), there is no actual evidence for this suggestion. Indeed, there is currently very little information available that allows inferring how (facultative or obligate) parthenogenesis evolved in any of the known mayfly species. Nevertheless, because of their low dispersal ability and their short and fragile adult life, mayflies have restricted opportunities for reproduction, which may frequently generate situations of mate limitation in females. Mate limitation has been shown to favor parthenogenesis in other insect species (Schwander et al. 2010), and is very likely to also select for parthenogenesis in mayflies, in spite of the probable trade-off with sexual reproduction we highlighted above.

### Fate of Sexual Traits in “Obligate” Parthenogenetic Mayflies

Sexual traits in asexual species decay more or less rapidly depending on whether they become costly or neutral upon transitions to parthenogenesis (reviewed in van der Kooi and Schwander 2014). Selection favors the reduction of costly traits, contrary to neutral traits that decay via drift. For example, sexual traits that could decay in parthenogenetic females are 1) pheromones, 2) the capacity to produce males, 3) the ability to fertilize their eggs, and 4) the synchrony of emergences with males.

Sex pheromones are chemical signals involved in mate choice (reviewed in Johansson and Jones 2007) that can disappear in asexual lineages (e.g., Schwander et al. 2013; Tabata et al. 2017). However, there are apparently no volatile pheromones in mayflies, with mate choice and species recognition based on visual signals and tactile recognition (Landolt et al. 1997; MS personal observation). Occasional production of males has been reported in a range of “obligately” parthenogenetic mayfly species (e.g., in *Ameletus ludens*Clemens 1922; Needham 1924; and in *Neocloeon triangulifer*Funk et al. 2006, 2010) similar to most parthenogenetic species in other animal groups (van der Kooi and Schwander 2014). In species with male heterogamety, male development in parthenogenetic lineages likely follows the accidental loss of an X-chromosome during oogenesis (Schwander et al. 2013). Accidental males produced by parthenogenetic females are often still able to fertilize eggs of females from sexual populations (van der Kooi and Schwander 2014), but there is currently little information on the fertility of accidental males in mayflies. If fertile, as shown for 2 baetid species (Funk et al. 2010), such males could potentially generate new lineages by matings with sexual females (i.e., contagious parthenogenesis as explained above), which could help explain the high frequency of parthenogenesis in mayflies.

The ability of parthenogenetic females to fertilize their eggs is unknown in mayflies overall, as only one species, the baetid *Neocloeon triangulifer*, has been studied thus far (Funk et al. 2006). In this species, the ability to fertilize eggs is maintained at least at low levels. Viable progeny could be obtained by crossing *Neocloeon alamance* males (a sexual species with XY male heterogamety) with parthenogenetic *N. triangulifer* females. In such crosses, 66.6% of offspring were normal, clonal *N. tringulifer* females with high fertility, suggesting they were produced parthenogenetically from unfertilized eggs. However, the remaining 33.3% were genetically intermediate between the 2 species (as indicated by allozyme genotypes), suggesting they were hybrids produced from fertilized eggs. Approximately half of this hybrid progeny were females, perhaps triploid, with low fertility, the second half consisted of sterile gynandromorphs (with both male and female morphological characteristics). These findings suggest that even “obligately” parthenogenetic mayfly species still produce haploid eggs, which could explain why there is always a small proportion of unfertilized eggs that never hatch (typically 3–22%), although more data are clearly needed.

Depending on how synchronous the emergences of both sexes are, the temporal windows to find a mate can be affected. Accordingly, obligate parthenogenesis might lead to a decay of the synchronized activity patterns in mayfly species. Tropical species of Trichoptera and Ephemeroptera appear to follow this theory (Tjønneland 1970). However, this does not hold for several other mayfly species from temperate regions (e.g., Ameletidae: *Ameletus ludens*; Baetidae: *Neocloeon triangulifer*; Ephemerellidae: *Ephemerella notata* and *Eurylophella funeralis*), where emergence patterns of parthenogenetic mayflies are at least as synchronous as for sexual species (Sweeney and Vannote 1982; Glazaczow 2001). These findings might indicate that synchronization of emergence is not costly in temperate regions, that there are other factors such as predation that select for the maintenance of emergence synchrony (Sweeney and Vannote 1982), or perhaps that there are still rare events of sexual reproduction even in species believed to be obligately parthenogenetic.

## Conclusion

We found evidence for parthenogenesis in at least 65 mayfly species, which represent 1.8% of the 3,666 described species. However, this frequency is likely underestimated given that among the 136 species whose reproductive mode was studied, this value reaches 47.8%, currently the highest estimate known in non-cyclical parthenogenetic organisms. Parthenogenesis in mayflies thus appears to be widespread and is certainly an order of magnitude more frequent than in animal groups surveyed thus far. Among the 71 mayfly species found to reproduce sexually, 38 (53.5%) can produce offspring by accidental parthenogenesis (i.e., tychoparthenogenesis). Such accidental parthenogenesis could function as a pre-adaptation for facultative parthenogenesis, which may often be selected in mayflies because their short adult life frequently generates situations of mate limitation.

We found that sex is a more successful strategy (associated with a higher hatching success of eggs) than parthenogenesis. Indeed, we found a trade-off between the hatching success of parthenogenetic and sexual eggs across mayfly species, meaning that improving the capacity for parthenogenesis may come at a cost for sexual reproduction, even in facultative parthenogens. Such a trade-off can help explain why facultative parthenogenesis is extremely rare among animals in spite of its potential to combine the benefits of sexual and parthenogenetic reproduction.

Additional studies focused on species from areas other than North America and Europe would be necessary to obtain a fully representative overview of the frequency of parthenogenesis in mayflies and for uncovering potential lineage-level or geographical-ecological correlates of parthenogenesis in this group. Additional studies are also needed regarding the cytological mechanisms and the origin of parthenogenesis in mayflies. In spite of these constraints, mayflies are currently clearly underappreciated for their value as outstanding model systems for testing the benefits of sex in natural populations.

## Supplementary Material

esaa027_suppl_Supplementary_Appendix

esaa027_suppl_Supplementary_Figures

## Data Availability

All data (Supplementary Appendix) are deposited in Dryad under https://doi.org/10.5061/dryad.fttdz08qf.
