# Supplementary material for: Extremely Widespread Parthenogenesis and a Trade-Off Between Alternative Forms of Reproduction in Mayflies (Ephemeroptera)
Source: J Hered. 2020 Sep 12;112(1):45–57. doi: 10.1093/jhered/esaa027 (PMC7953839; doi:10.1093/jhered/esaa027)
Supplement: esaa027_suppl_Supplementary_Figures [file esaa027_suppl_supplementary_figures.docx]

**Supplementary Materials**


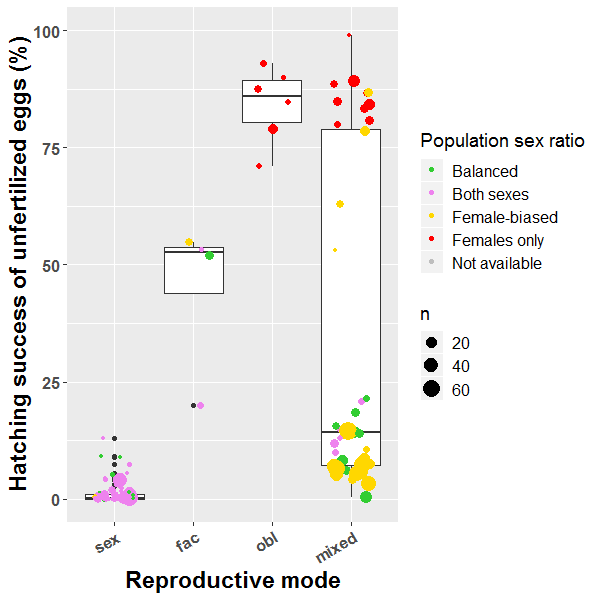


**Supplementary Figure 1**. Population-average hatching success of unfertilized eggs and reproductive modes (**without the Baetidae family**). x axis: sex: sexual reproduction, fac: facultative parthenogenesis, obl: “obligate” parthenogenesis, mixed: mixed reproduction (in sympatry and/or in allopatry). Population sex ratio with both sexes (purple dots) means that males are present in these populations but exact sex ratios were not recorded; *n*: number of females used for determining the egg-hatching success in a given population. In total, 126 populations from 75 different species are represented (with species from 16 families but mostly Heptageniidae (40.5%), see also Supplementary Figure 2).


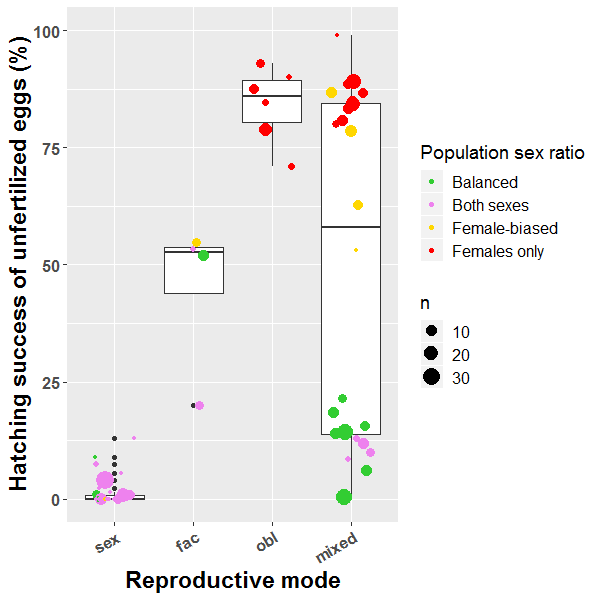


**Supplementary Figure 2**. Population-average hatching success of unfertilized eggs and reproductive modes (**without the Baetidae and Heptageniidae families**). x axis: sex: sexual reproduction, fac: facultative parthenogenesis, obl: “obligate” parthenogenesis, mixed: mixed reproduction (in sympatry and/or in allopatry). Population sex ratio with both sexes (purple dots) means that males are present in these populations but exact sex ratios were not recorded; *n*: number of females used for determining the egg-hatching success in a given population. In total, 75 populations from 52 different species are represented (among 15 families).


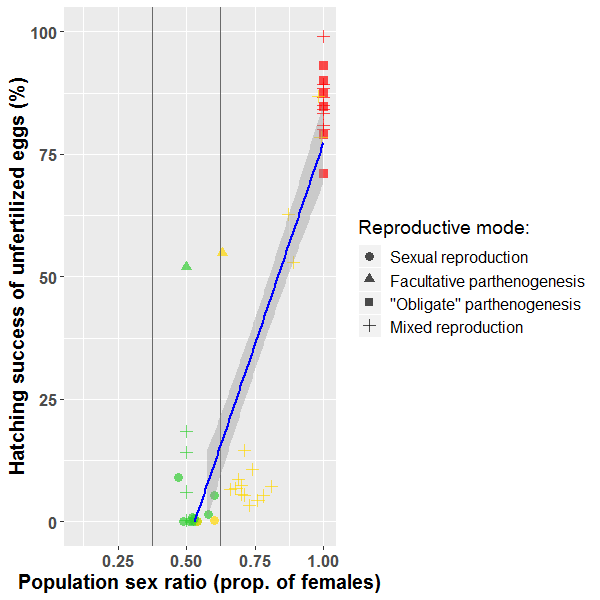


**Supplementary Figure 3**. Population-level correlation between unfertilized egg-hatching success and sex ratio (**without the Baetidae family**; *GLMM*, *r = 0.88, P-value < 0.001*). Data are available for 49 populations from 18 species, among 10 families (but mostly Heptageniidae (36.7.2%), see also Supplementary Figure 4).


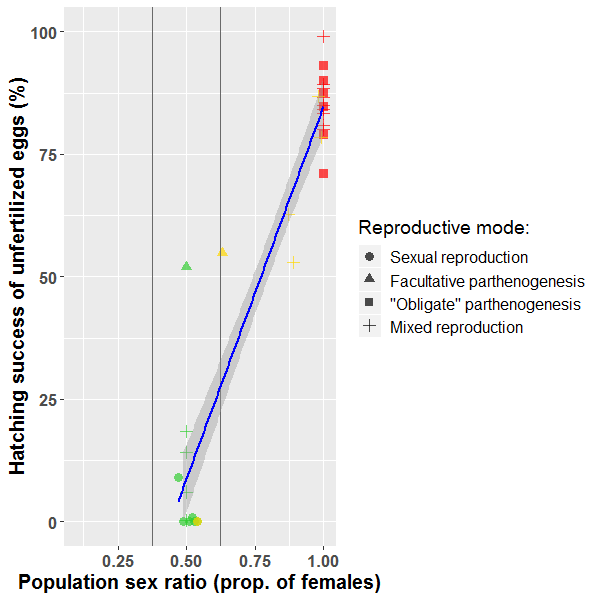


**Supplementary Figure 4**. Population-level correlation between unfertilized egg-hatching success and sex ratio (**without the Baetidae and Heptageniidae families**; *GLMM, r = 0.97, P-value < 0.001*). Data are available for 32 populations from 14 species, among 9 families.


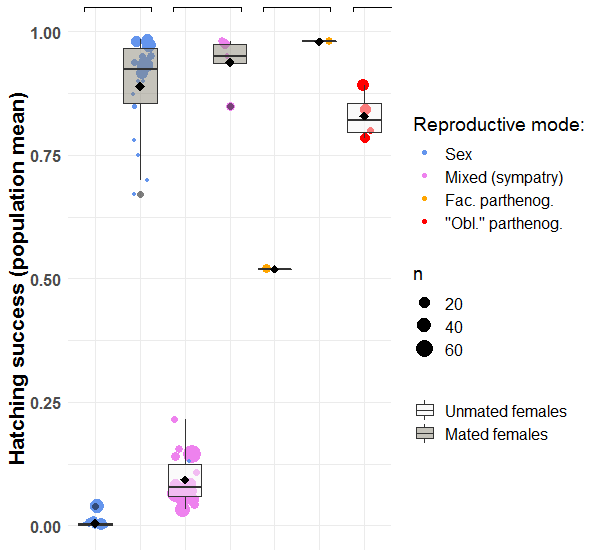


**Supplementary Figure 5.** Hatching success of fertilized and unfertilized eggs for species with different reproductive modes and mating status (**without the Baetidae family**). Data are available for 65 populations from 19 different species, among 7 families (but mostly Heptageniidae (35.4%), see also Supplementary Figure 6). *n*: number of females tested for a given population. Sex: sexual reproduction (*P < 0.001*), Mixed: mixed reproduction in sympatry (*P < 0.001*), Fac. parthenog.: facultative parthenogenesis (*P = 0.053*), “Obl.” parthenog.: “obligate” parthenogenesis.

**
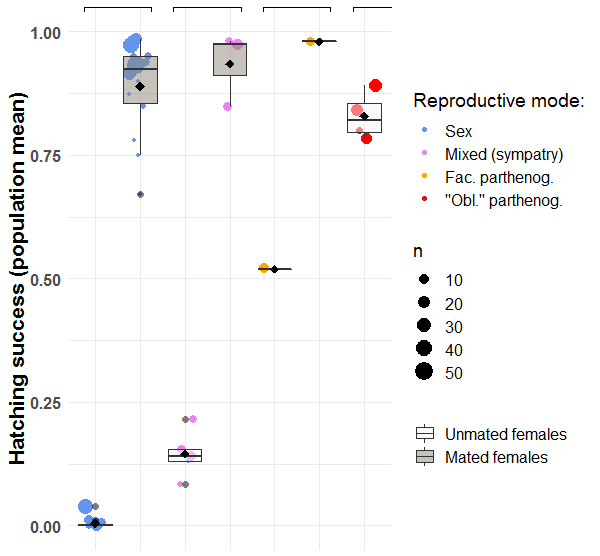
**

**Supplementary Figure 6.** Hatching success of fertilized and unfertilized eggs for species with different reproductive modes and mating status (**without the Baetidae and Heptageniidae families**). Data are available for 42 populations from 14 different species, among 6 families. *n*: number of females tested for a given population. Sex: sexual reproduction (*P < 0.001*), Mixed: mixed reproduction in sympatry (*P < 0.001*), Fac. parthenog.: facultative parthenogenesis (*P = 0.003*), “Obl.” parthenog.: “obligate” parthenogenesis.
